# A recurrent *F8* mutation (c.6046C>T) causing hemophilia A in 8% of northern Italian patients: evidence for a founder effect

**DOI:** 10.1002/mgg3.189

**Published:** 2015-12-14

**Authors:** Isabella Garagiola, Sabrina Seregni, Mimosa Mortarino, Maria Elisa Mancuso, Maria Rosaria Fasulo, Lucia Dora Notarangelo, Flora Peyvandi

**Affiliations:** ^1^ Angelo Bianchi Bonomi Haemophilia and Thrombosis CenterFondazione IRCCS Cà Granda Ospedale Maggiore PoliclinicoUniversità degli Studi di Milano and Luigi Villa FoundationMilanItaly; ^2^ Pediatric Onco‐Hematology and BMT UnitChildren HospitalSpedali CiviliBresciaItaly

**Keywords:** *F8* gene, founder effect, hemophilia A, haplotype analysis, recurrent mutation

## Abstract

Hemophilia A is a heterogeneous hemorrhagic disorder caused by a large number of mutations. Recurrent mutations are rare, except intron 22 and intron 1 inversions. The substitution of a cytosine to a thymine at nucleotide 6046 in *F8* gene was identified in a group of Italian patients affected by hemophilia A from a specific region of Northern Italy with a prevalence of 7.6%. This *F8* variant was the second most frequent mutation in our cohort, after the intron 22 inversion. The identification of the same mutation in a restricted population gets to suppose the existence of a founder effect. Intragenic and extragenic polymorphic markers were tested to assess this assumption. A peculiar haplotype in linkage disequilibrium with this recurrent mutation (c.6046C>T) was identified in 71% of patients, supporting a founder effect. This distinctive haplotype was not identified in a control group (Fisher's exact test, *P* < 0.0001), coming from the same geographic region. These data strongly suggested the presence of a founder effect, supporting the existence of a single mutation event. Using DMLE+2.3 software and the mathematical approach described by Bengtsson and Thomson, the inferred age of this mutation is supposed to be about 2325 years (95% CI: 904–5081) ago.

## Introduction

Up to date, more than 100 X‐linked inherited human disorders or traits have now been identified, most of which are classified as recessive (McKusick 1998). Among the human X‐linked recessive diseases, hemophilia A (HA; MIM #306700) represents the most common hereditary bleeding disorder, determined by decreased factor VIII (FVIII) levels in plasma with a proportional reduction in FVIII clotting activity (FVIII:C). FVIII is a plasma glycoprotein with an essential role in the intrinsic coagulation pathway, hence a deficiency or dysfunction of FVIII causes limited and delayed generation of thrombin, with defects in clot formation and consequent bleeding diathesis. On the basis of residual FVIII:C, hemophilia is categorized in three main forms: severe (FVIII:C < 1 IU/dL), moderate (FVIII:C 1–5 IU/dL), and mild (FVIII:C 6–39 IU/dL) (Mannucci and Tuddenham 2001). The clinical picture of severe hemophilia patients is characterized by frequent spontaneous bleeding episodes that commonly occur into joints and muscles. Recurrent hemarthroses may cause in these patients crippling arthropathy on the long‐term if not properly treated (Mannucci and Tuddenham 2001).

The primary structure of FVIII was identified in the early 1980s, when the protein was purified and its cDNA has been cloned (Gitschier et al. 1984; Vehar et al. 1984). A few years later, in 1986 by in situ hybridization experiments, the cytogenetic localization of the *F8* gene (MIM #300841; NC_000023.11) has been displayed in the proximal part of the long arm of the X chromosome (Xq28) (Tantravahi et al. 1986). FVIII is synthesized as a single polypeptide chain, containing 2332 amino acids arranged within six domains organized as A1‐A2‐B‐A3‐C1‐C2. The molecule circulates in plasma as a heterodimer (~300 kDa) composed of the two polypeptide chains. (Vehar et al. 1984). The light chain, assembled by A3‐C1‐C2 domains and a heterogeneous heavy chain consisted of A1‐A2‐B domains. Immediately after release into circulation, FVIII forms a tight noncovalent complex with von Willebrand Factor (VWF) and the complex formation allows to maintain physiological FVIII levels in the circulation (Lenting et al. 1998).

Hemophilia A is a heterogeneous genetic disease associated with a large number of mutations. The most common mutation, which occurs in 45–50% of patients with severe hemophilia A, is the intron 22 inversion caused by an intrachromosomal homologous recombination between a 9.5‐kb region in intron 22 (*int22h‐1*) and either of two extragenic, distal homologous, *int22h‐2* and *int22h‐3* during meiosis (Lakich et al. 1993). Another common *F8* gene defect is the intron 1 inversion that causes 1–6% of all severe cases of hemophilia A (Bagnall et al. 2002).

The worldwide Factor VIII Variant Database, (http://factorviii-db.org/), reports and includes 2015 unique mutations distributed uniformly across the entire peptide chain of FVIII (updated October 2015). Point mutations (missense, nonsense, and splice site mutations) represent approximately 70% of the described molecular defects in hemophilia A.

By screening hemophilia A patients followed at the Angelo Bianchi Bonomi Haemophilia and Thrombosis Center (Milan, Italy) (from 1998 to 2014), a recurrent point mutation was identified in a large number of patients coming from a specific region of Northern Italy. The identification of the same mutation in patients from a well‐identified geographic region supports the hypothesis of a founder effect. In this study, we address the question whether this recurrent point mutation had a single origin and, if so, when the mutational event had occurred. To this end, we used the observed distribution of allelic variants on disease and control chromosomes to estimate the age of the mutation.

## Materials and Methods

### Subjects

A total of 364 patients from 317 not related families with moderate/severe hemophilia A referred to the Angelo Bianchi Bonomi Haemophilia and Thrombosis Center (Milan, Italy) were recruited. From the same area of Italy, 96 subjects were recruited as control group. All subjects involved gave their written informed consent according to the Helsinki Declaration.

### DNA extraction and FVIII gene analysis

DNA was extracted from leukocytes using standard salting‐out procedure (Miller and Djkes 1988). Intron 22 inversion gene rearrangement was detected by long‐range PCR and performed as previously described (Liu et al. 1998). Intron 1 inversion detection was done by PCR, as reported earlier (Bagnall et al. 2002). Coding regions, intron/exon boundaries, and the 5′ and 3′ untranslated regions of the *F8* gene (NG_011403.1) were amplified by PCR and sequenced by Sanger method using the ABI Prism BigDye^™^ Terminator Cycle Sequencing Ready Reaction Kit (Applied Biosystem, Foster City, CA) on an ABI PRISM 3130^™^ Genetic Analyzer (Applied Biosystems). Data analysis was carried out with Software Sequencing Analysis v3.0 (AB Applied Biosystems). Sequence alignment was done using Basic Local Alignment Search Tool (http://blast.ncbi.nlm.nih.gov). All oligonucleotides and PCR conditions are available on request.

### Nomenclature

Nucleotides are numbered from the first adenine in the ATG initiator methionine codon, and amino acids are numbered from 1 to 2351, starting with the first methionine as +1. The mutations are reported following the guidelines of the Human Genome Variation Society (http://www.hgvs.org/mutnomen/recs.html).

### Microsatellite analysis

**Figure 1:**
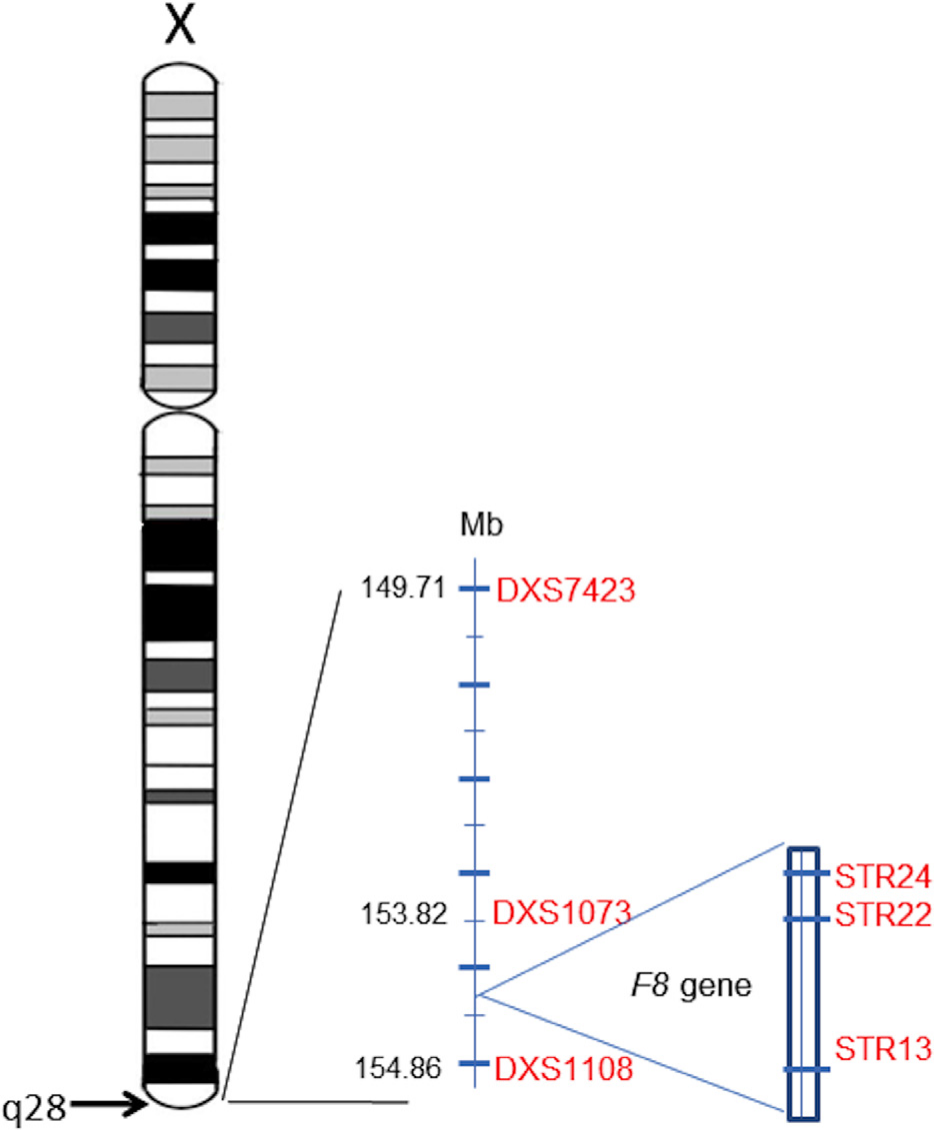
Genomic map of chromosome X: localization and position of *F8* gene and polymorphic markers on Xq28.

For haplotype analysis, six polymorphic microsatellite markers have been selected. Three markers are within intron 13, 22, and 25 (STR13, STR22, and STR24) (Sanchez‐Garcia et al. 2005; Liang et al. 2009) in *F8* and three extragenic markers (DXS7423, DXS1073, and DXS1108) (Edelmann et al. 2002; Sanchez‐Garcia et al. 2005; Fimiani et al. 2006) flanking *F8* gene. All microsatellite markers are located in the same chromosome X region, Xq28 (Fig. 1). Markers details are described in Table 1.

**Table 1:** Position (Mb), type of repeat, fragment length (bp) of sequence PCR fragment, numbers of alleles and heterozygosity rate (HR) of extragenic and intragenic polymorphic markers

UniSTS	Marker	Xq band	Position (Mb)	Type of repeat	Fragment length (bp)	Alleles (n)	Heterozygosity rate (HR) %
99583	DXS7423	Xq28	149.71	TCCA	175–199	7	N.A.
53337	DXS1073		153.82	TG	120–146	8	70.7
	STR24		154.07	GT	178–196	5	38
	STR22		154.10	GT	198–210	4	46.3
	STR13		154.16	TG	148–164	6	46.3
147826	DXS1108		154.86	CA	160–177	6	65.9

Polymerase chain reaction (PCR) was performed using fluorescent end‐labeled PCR primers, a HEX‐labeled oligonucleotide for DXS1073, a FAM‐labeled oligonucleotide for DXS1108, DXS7423, and STR22, and AT565‐labeled oligonucleotide for STR13 and STR24. PCR products were analyzed by capillary electrophoresis in an ABI PRISM 3130^™^ Genetic Analyzer (Applied Biosystems). Sizing was performed with ABI GeneMapper 4.0 (Applied Biosystems).

### Allelic association and linkage analyses

Differences in allele proportions between disease and normal chromosomes were analyzed using *Z*‐test to determine whether the differences observed were significant. The probability associated with the *Z*‐score was assessed and the statistical threshold was set at 0.05.

To evaluate the allelic association between the mutation and the original associated loci, the degree of linkage disequilibrium (*δ*) was assessed as defined by Bengtsson and Thomson *δ* = (p_D_ − p_N_)/(1 − p_N_) (Bengtsson and Thomson 1981), where p_D_ is the frequency of the associated allele on disease chromosomes and p_N_ the frequency of the same allele on normal chromosomes. The linkage analysis was performed using the LOD score test developed by Newton E. Morton (Morton 1955) and described in detail by Strachan and Read. The LOD score test compares the likelihood of obtaining the test data if the two loci are linked (*θ* < 0.5), to the likelihood of observing the same data if the two loci are unlinked (*θ* = 0.5). Positive LOD scores favor the presence of linkage, whereas negative LOD scores indicate that linkage is less likely. By convention, a LOD score >3.0 is considered evidence for linkage, as it indicates 1000 to 1 odds that the linkage being observed did not occur by chance. On other hand, a LOD score less than −2.0 is considered evidence to exclude linkage. The LOD score is calculated as follows LOD = *Z* = log_10_(1 − *θ*)NR(*θ*)*R*/0.5(NR + *R*) where NR denotes the number of nonrecombinant offspring, and *R* denotes the number of recombinant offspring. A series of LOD scores are obtained using different linkage distances, and the maximum likelihood was estimated for the recombination fraction *θ*_max_.

### Estimation of the mutation age

Estimation of the c.6046C>T mutation age was carried out using the DMLE+2.3 software (Reeve and Rannala 2002) (http://www.dmle.org/). This software uses the Markov chain Monte Carlo algorithm for Bayesian inference of mutation age based on the observed linkage disequilibrium at multiple genetic markers. The estimation of the mutation age is based on the following parameters: the observed haplotypes (or genotypes) in subjects with a specific mutation and in normal subjects, map distances among markers and position of the mutation relative to markers, the estimated population growth rate (*r*), and an estimate for the proportion of mutation‐bearing chromosomes (*f*).

A second mathematical approach, described by Bengtsson and Thomson (Bengtsson and Thomson 1981), was used to confirm our findings. This is a moment method based on the linkage disequilibrium (*δ*), and the recombination frequency (*θ*), as expressed in the algorithm ĝ = log *δ*/log(1 − *θ*), where ĝ is the age of the mutation expressed as the number of generations. The Luria–Delbrück correction was applied to this method to avoid the risk of underestimation (Luria and Delbruch 1943).

## Results

A total of 364 patients from 317 not related families with moderate/severe hemophilia A were first screened for common *F8* inversions. In this patient population, the prevalence of intron 22 inversion and intron 1 inversion was 33.4% (106/317) and 1.9% (6/317), respectively. Inversion‐negative samples (64.7%, 205/317) were characterized by direct sequencing of all coding regions and intron‐exon boundaries of the *F8*.

**Figure 2:**
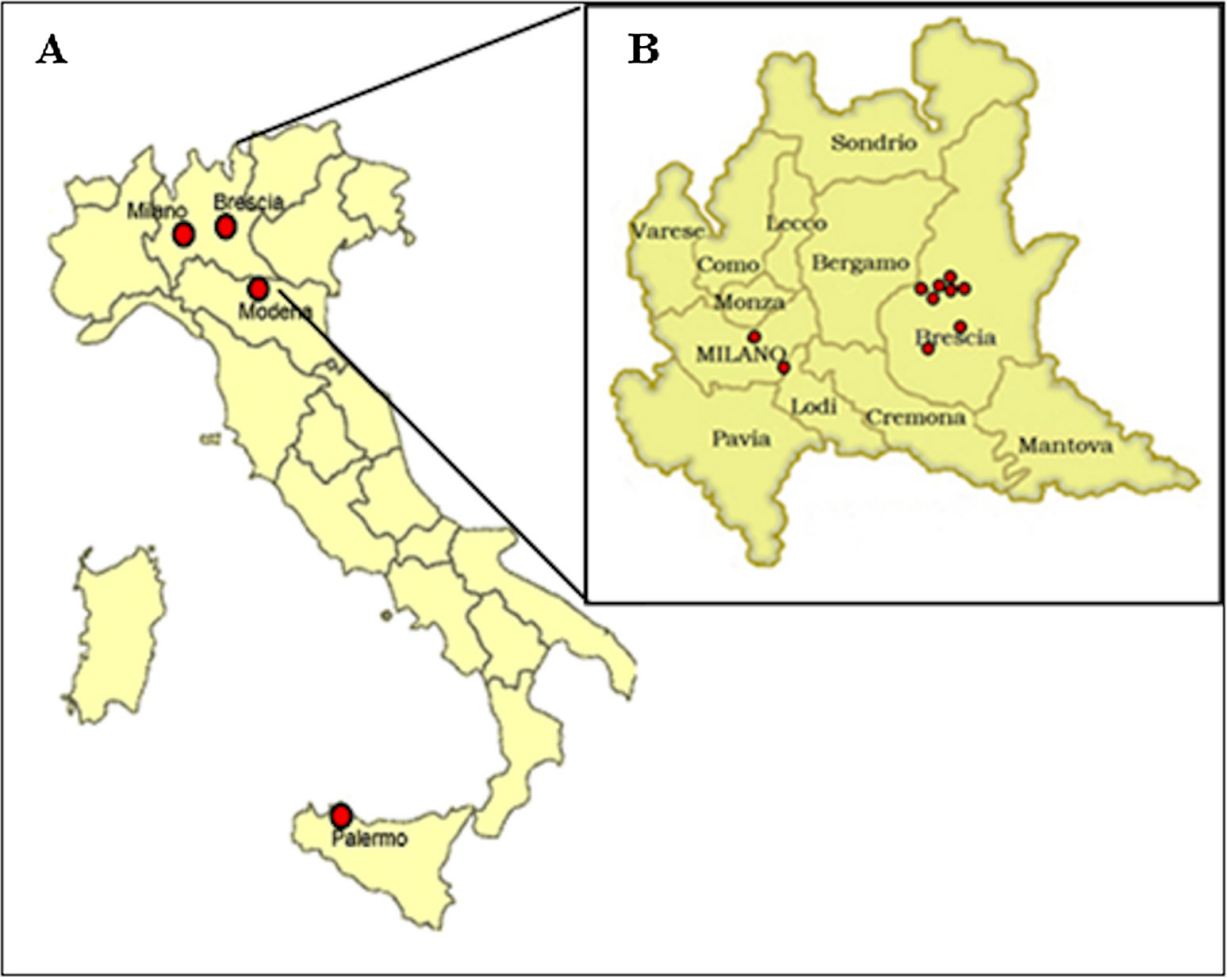
(A) Geographic distribution of patients carrying the mutation c.6046C>T in Italy. (B) Geographic distribution of patients carrying the mutation c.6046C>T in Lombardy.

A recurrent point mutation c.6046C>T (NM_000132.3; p.R2016W, NP_000123.1) was identified in 45 patients belonging to 24 not related families (7.6%, 24/317) from different Italian regions. Twenty‐two families came from Lombardy region (21 from Brescia, one from Milan), one from Emilia Romagna, and one from Sicily (Fig. 2). Patients carrying the c.6046C>T substitution have shown variable FVIII:C ranging from <1 to 6 IU/dL.

### Microsatellites analysis

To evaluate the hypothesis of a founder effect in our patients carrying the recurrent mutation c.6046C>T, a haplotype analysis was performed in all unrelated patients (*n* = 24) and in a control group without the recurrent mutation (*n* = 96). Five different haplotypes were observed in 24 unrelated patients (Table 2). A peculiar haplotype, defined as H1, characterized by followed alleles 189, 128, 190, 206, 156, 171, was identified in the 71% (17/24) of patients with the recurrent mutation. An additional haplotype (H2) differed from H1 for the most distal marker, DXS7423, was identified in 4/24 (17%) patients. All patients with H1 and H2 haplotype (21/24, 87.5%) were from specific area close to Brescia, a city in Lombardy region, Northern of Italy. Haplotype H3 differed from H1 for two extragenic markers, DXS1073 and DXS7423, and was identified in only one patient originating from Palermo. Haplotype H4 was similar to the ancestral haplotype H1 for two extragenic markers DXS1108 and DXS7423, while H5 was totally different from ancestral haplotype.

**Table 2:** Haplotypes flanking the c.6046C>T mutation (NM_000132.3) in 24 Italian patients

Haplotype	DXS7423	DXS1073	STR24	STR22	c.6046C>T	STR13	DXS1108	Frequency
H1	189	128	190	206	T	156	171	17/24 (71%)
H2	185	128	190	206	T	156	171	4/24 (17%)
H3	177	130	190	206	T	156	171	1/24 (4%)
H4	189	142	196	204	T	158	171	1/24 (4%)
H5	181	144	194	204	T	154	161	1/24 (4%)

None of the haplotypes associated with the c.6046C>T mutation were present in 96 controls, coming from the same geographic region (Fisher's exact test, *P* < 0.0001).

### Allelic association and linkage analyses

We analyzed also the frequencies of each marker allele associated with the c.6046C>T and we compared them with the corresponding allele frequency in the control group (Table 3). The three alleles, 189, 128, and 171, corresponding to extragenic markers DXS7423, DXS1073, and DXS1108, respectively, and contributing to the ancestral haplotype on the c.6046C>T chromosomes, occurs at significantly increased frequency (*P* < 0.0001). A significant difference in allele proportions, between patients and controls, also emerged for the allele 156 corresponding to intragenic marker STR13, while not significant difference emerged for alleles 206 and 190 associated to intragenic marker STR22 and STR24, respectively.

**Table 3:** Linkage Disequilibrium between the c.6046C>T *F8* mutation and markers in Xq28

Marker (allele)	Ancestral allele proportions^a^		Difference in proportions		Linkage disequilibrium (*δ*)^b^
	*p*_*D*_	*p*_*N*_	95% CI_Δp_	*P* value	95% CI_*δ*_
DXS7423 (189)	0.750 (18/24)	0.156 (15/96)	0.594 (95% CI: 0.405–0.781)	<0.0001	0.704 (95% CI: 0.507–0.901)
DXS1073 (128)	0.875 (21/24)	0.167 (16/96)	0.708 (95% CI: 0.556–0.860)	<0.0001	0.850 (95% CI: 0.692–1.000)
STR24 (190)	0.917 (22/24)	0.833 (80/96)	0.083 (95% CI: −0.050–0.216)	0.306	0.500 (95% CI: 0.362–0.638)
STR22 (206)	0.917 (22/24)	0.656 (63/96)	0.260 (95% CI: 0.114–0.407)	0.012	0.758 (95% CI: 0.606–0.909)
STR13 (156)	0.917 (22/24)	0.500 (48/96)	0.417 (95% CI: 0.267–0.566)	<0.0001	0.833 (95% CI: 0.678–0.988)
DXS1108 (171)	0.958 (23/24)	0.302 (29/96)	0.656 (95% CI: 0.534–0.778)	<0.0001	0.940 (95% CI: 0.815–1.000)

a pD and pN are the frequencies of the marker allele on disease‐mutation bearing and normal chromosomes, respectively.

b Calculated according to the method of Bengtsson and Thomson: *δ *= (pD − pN)/(1 − pN).

The values of the linkage disequilibrium parameter (*δ*) for the six markers are given in Table 3. Considering the intragenic markers, higher values of *δ* were observed at STR13 (allele 156, *δ* = 0.833) and at STR22 (allele 206, *δ* = 0.758) loci than at STR24 (allele 190, *δ* = 0.500). Whereas, *δ* is higher at the most proximal extragenic locus DXS1108 (allele 171, *δ* = 0.940) and decreases slightly at DXS1073 (allele 128, *δ* = 0.850), whereas it is lower at the most distal locus DXS7423 (allele 189, *δ* = 0.704).

The LOD score analysis was performed between the cluster of the three intragenic markers (STR24, STR22, STR13), assumed as single locus, and between the extragenic markers DXS7423, DXS1073, and DXS1108. LOD scores for each locus using different linkage distances (*θ*) and the estimate of the maximum likelihood for the recombination fraction *θ*_max_ are reported in Table 4. The intragenic locus STR24/STR22/STR13 showed the highest LOD score (12.69 at *θ* = 0.08), the most proximal extragenic locus DXS1108 showed a maximum LOD score of 5.41 at *θ* = 0.04, which decreased for DXS1073 (3.29 at *θ* = 0.12) and was lower for the most distal locus DXS7423 (1.36 at *θ* = 0.25) (Table 4). Higher values of LOD score were obtained with the cluster of the three intragenic markers and with the proximal telomeric marker (DXS1108) indicating no cross over occurred.

**Table 4:** Maximum LOD SCORES between the c.6046C>T *F8* mutation and markers in Xq28

Marker	*θ* a_max_	Maximum LOD SCORE^b^
DXS7423	0.25	1.36
DXS1073	0.12	3.29
STR13/STR22/STR24	0.08	12.69
DXS1108	0.04	5.41

a *θ *< 0.5.

b LOD = Z = log10 (1 − *θ*)NR(*θ*)*R*/0.5(NR + *R*) where NR and *R* are the number of nonrecombinant and recombinant chromosomes, respectively.

LOD score >3 is evidence for linkage.

LOD score <−2 is evidence to exclude linkage.

### Estimation of the c.6046C>T mutation age

The DMLE+ software requires an estimation of the population growth rate (*r*) and the proportion of disease‐bearing chromosome (*f*). The population growth rate (*r*) was estimated by the following equation, as previously described^1^: *T*_1_ = *T*_0_e^(gr)^, in which *T*_1_ is the estimated size of Italian population today (~60 million at 2012), T_0_ is the estimated size of the ancestral population (~3 million at the beginning of 5th century before Christ), and *g* is the number of generations between these two time points (*g *=* *100.48 considering 25 years for a generation). Accordingly, population growth rate was estimated to be approximately equal to 0.03. Using a previously described method (Borroni et al. 2011), the proportion of disease‐bearing chromosomes was calculated by estimating the frequency of the c.6046C>T mutation in the Italian male population (28 million): considering the frequency of hemophilia A (1:5000 male births) and giving a mutation frequency of 7.6% (24/317), the proportion of disease‐bearing chromosomes was estimated to be 0.06.

**Figure 3:**
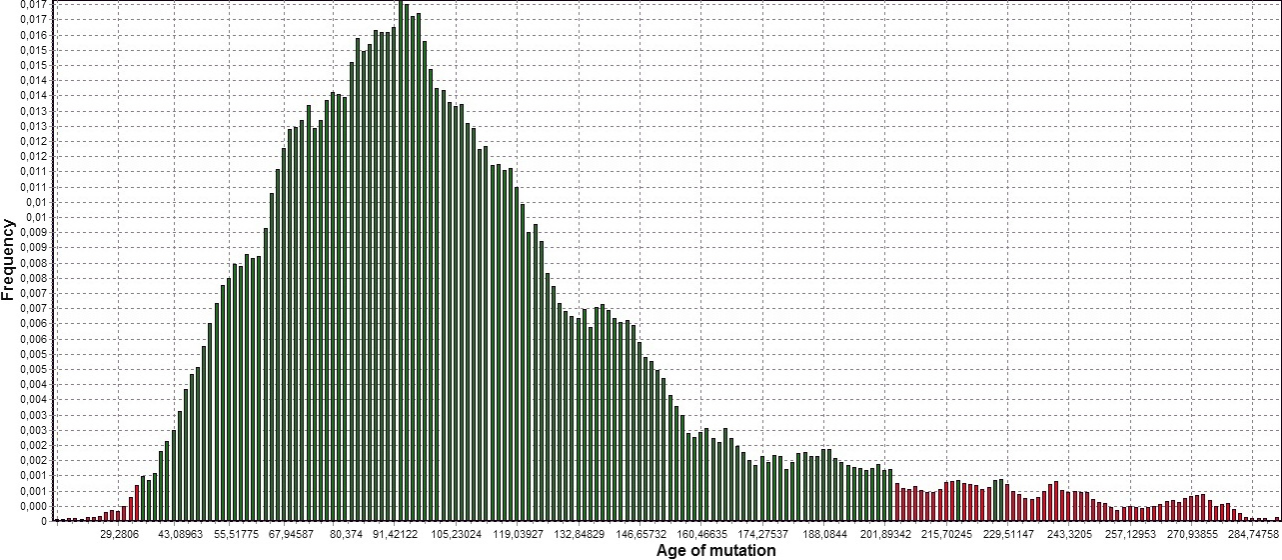
Age estimation of c.6046C>T mutation calculated by the software DMLE+2.3. Distribution of the posterior probability as a function of the age of c.6046C>T, assuming 0.06 as the proportion of disease‐bearing chromosomes (f) and 0.03 as population growth parameter (*r*). In the graph ‘Age of mutation’ is expressed as ‘number of generation’.

Assuming that *r *=* *0.03 and *f *=* *0.06, analysis results showed that the mutation c.6046C>T could be originated 93 generations ago (95% CI: 36–203), and considering generations of 25 years, these data indicate that the c.6046C>T mutation could be originated 2325 years ago (95% CI: 904–5081), among 300 years before the common era (BCE) (Fig. 3).

When the mathematical algorithm ĝ = log*δ*/log(1 − *θ*) (Bengtsson and Thomson 1981), was used and the Luria–Delbrück correction (Luria 1945) was applied, assuming that *r *=* *0.03, the age of the mutation c.6046C>T was estimated to be from 56 to 128 generations (1387–3200 years) (Table 5).

**Table 5:** Linkage disequilibrium analysis and age calculation for the c.6046C>T mutation

Marker (allele)	Physical position (Mb)	Distance from c.6046C>T		LD	Estimated age	
		Mb	*θ* a	*δ* b	g^c^	Years^d^
DXS7423 (189)	149.71	4.42	0.0442	0.704	**–**	**–**
DXS1073 (128)	153.82	0.31	0.0031	0.850	128.00	3200
STR24 (190)	154.07	0.06	0.0006	0.500	**–**	**–**
STR22 (206)	154.10	0.03	0.0003	0.758	**–**	**–**
c.6046C>T	154.13	**–**	**–**	**–**	**–**	**–**
STR13 (156)	154.16	0.03	0.0003	0.833	**–**	**–**
DXS1108 (171)	154.86	154.86	1.5486	0.940	55.51	1387

a Assuming Mb~cM.

b Calculated according to the method of Bengtsson and Thomson: *δ *= (pD − pN)/(1 − pN).

c ĝ = log*δ*/log(1 − *θ*); Luria–Delbrück correction *g* = −(1/*r*)ln(*θ*fr), assuming *r* = 0.03 and fr = 1/*r*.

d Assuming generations lasting 25 years.

## Discussion

We identified a mutation of *F8* (c.6046C>T) that is the second most frequent in our patients affected by hemophilia A, after the intron 22 inversion with a prevalence of 7.6%. This variant was identified in a group of patients coming from a specific area close to Brescia, a city in Lombardy region of Northern Italy. The high prevalence of the mutation (c.6046C>T) in a restricted Italian population is consistent with previous findings indicating that the disease may be transmitted by founder effect in stable populations with low inward migration (Winter et al. 2008). Haplotype analysis using intragenic and extragenic microsatellites markers was employed to investigate the hypothesis of a potential founder effect. A common haplotype (H1), covering over 5.15 Mb in the region Xq28 including *F8* gene, was found in 71% of unrelated patients with the recurrent mutation. An additional haplotype (H2) different for the most distal marker (DXS7423), was present in 17% of patients. This haplotype could probably be originated from the ancestral and this could be explained by recombination events. Indeed, the linkage parameters (*θ*_max_ and LOD score) of this marker demonstrated the evidence of a higher likelihood for the recombination event. However, it cannot be excluded that some modifications could have originated by slippage mispairing at the most distal marker DXS7423. Patients sharing the H1 and H2 haplotype were from a specific area close to Brescia and these combination of marker alleles was not detected in any of the 96 Italian subjects belong from the same Italian region of patients (Fisher's exact test, *P *<* *0.0001).

The difference between the haplotype distribution in patients and controls was confirmed when single marker alleles associated with the mutation were analyzed, since their frequency in patients was significantly higher than that in controls, except for STR24 (allele 190). This allele frequency was similar both in patients and controls, according to low heterozygosity rate of STR24 in the general population.

The region extended from DXS1108 to DXS7423 markers resulted to be in linkage disequilibrium showing a statistical association between the mutation and the six alleles. The presence of a large region in linkage disequilibrium could be a result of founder effect and decreased allelic diversity, typical features of isolated and stable populations. In addition, LOD score analysis gave an evidence of linkage between the mutation and the associated loci. We can suppose that unrelated people with the disease have actually inherited it from a distant common ancestor, thus they tend to share particular ancestral alleles at loci closely linked to the mutation locus. Moreover, two haplotypes (H4 and H5) were found in patients coming from other regions of Italy. Since the patients with the ancestral haplotypes (H1) originate from one restricted geographic area surrounding Brescia, we assume that they represent a relatively isolated population with an extensive linkage disequilibrium than outbred population. Then, the difference between haplotypes H4 and H5 compared to ancestral may arise from the diverge between populations over the course of many generation or contrariwise that the mutation arose later in a different population. To further evaluate whether the recurrent mutation had a single origin and, in case, when the mutational event has been occurred, an attempt to estimate time to the origin of the mutation was performed by the DMLE method and the mathematical algorithm of Bengtsson and Thomson. Based on the DMLE analysis, the c.6046C>T mutation was estimated to be approximately 96 generations old, about 2100 years old assuming 25 years per generation, and the mathematical algorithm gave a similar result. These estimates place the origin of the mutation during the II century before Christ (BC), when north Italy was experiencing an heavy influence from Celtic populations that, from 400 BC, descended in North Italy and occupied Padana plain. Under Roman Empire, from II century BC, Brescia, located in Padana plain, became the most important center of tribe of Galli (as roman called Celts) Cenomani that reached also pre‐Alpine mountains surrounding Brescia establishing their tribe culture and maintaining their autonomy from Romans.

Since almost all patients carrying the mutation originated from Brescia and showed a prevalent haplotype associated with the mutation, it is tempting to speculate that the common ancestor lived in a relatively isolated area of Brescia, perhaps pre‐Alpine area of Valtrompia, from which the majority of patients came from. Thus, it is plausible that the mutation frequency increased as a result of a local founder effect and that low migratory fluxes for long time favored the expansion of the mutation in this area.

Furthermore, we cannot exclude that this mutation might have spread far from Brescia due to more recent migrations. It would be interesting to see whether the other patients reported in Europe, Asia, America descend from the same ancestor. Analysis of additional populations is required to definitely determine the origin and the age of the ancestor allele.

## Conflict of Interest

None declared.

## References

[mgg3189-bib-0001] Bagnall, R. D. , N. Waseem , P. M. Green , and F. Giannelli . 2002 Recurrent inversion breaking intron 1 of the factor VIII gene is a frequent cause of severe Haemophilia A. Blood 99:168–174. 11756167 10.1182/blood.v99.1.168

[mgg3189-bib-0002] Bengtsson, B. O. , and G. Thomson . 1981 Measuring the strength of associations between HLA antigens and diseases. Tissue Antigens 18:356–363. 7344182 10.1111/j.1399-0039.1981.tb01404.x

[mgg3189-bib-0003] Borroni, B. , C. Bonvicini , D. Galimberti , L. Tremolizzo , A. Papetti , S. Archetti , et al. 2011 Founder effect and estimation of the age of the Progranulin Thr272fs mutation in 14 Italian pedigrees with frontotemporal lobar degeneration. Neurobiol. Aging 32:555–558. 20947212 10.1016/j.neurobiolaging.2010.08.009

[mgg3189-bib-0004] Edelmann, J. , D. Deichsel , S. Hering , I. Plate , and R. Szibo . 2002 Sequence variation and allele nomenclature for the X‐linked STRs DXS9895, DXS8378, DXS7132, DXS6800, DXS7133, GATA172D05, DXS7423 and DXS8377. Forensic Sci. Int. 129:99–103. 12243877 10.1016/s0379-0738(02)00230-x

[mgg3189-bib-0005] Fimiani, G. , C. Laperuta , G. Falco , V. Ventruto , M. D'Urso , M. V. Ursini , et al. 2006 Heterozygosity mapping by quantitative fluorescent PCR reveals an interstitial deletion in Xq26.2‐q28 associated with ovarian dysfunction. Hum. Reprod. 21:529–535. 16239311 10.1093/humrep/dei356

[mgg3189-bib-0006] Gitschier, J. , W. I. Wood , T. M. Goralka , K. L. Wion , E. Y. Chen , D. H. Eaton , et al. 1984 Characterization of the human factor VIII gene. Nature 312:326–330. 6438525 10.1038/312326a0

[mgg3189-bib-0007] Lakich, D. , H. H. Jr Kazazian , S. E. Antonarakis , and J. Gitschier . 1993 Inversions disrupting the factor VIII gene are a common cause of severe haemophilia A. Nat. Genet. 5:236–241. 8275087 10.1038/ng1193-236

[mgg3189-bib-0008] Lenting, P. J. , J. A. van Mourik , and K. Mertens . 1998 The life cycle of coagulation factor VIII in view of its structure and function. Blood 92:3983–3996. 9834200

[mgg3189-bib-0009] Liang, Y. , Y. Zhao , M. Yan , X. P. Fan , B. Xiao , and J. Z. Liu . 2009 Prenatal diagnosis of haemophilia A in China. Prenat. Diagn. 29:664–667. 19399824 10.1002/pd.2271

[mgg3189-bib-0010] Liu, Q. , G. Nozari , and S. S. Sommer . 1998 Single‐tube polymerase chain reaction for rapid diagnosis of the inversion hotspot of mutation in Haemophilia A. Blood 92:1458–1459. 9694739

[mgg3189-bib-3000] Luria, SE , M. Delbruch . 1943 Mutations of bacterial from virus sensitivity to virus resistance. Genetics 28:491‐511. 17247100 10.1093/genetics/28.6.491 PMC1209226

[mgg3189-bib-0011] Luria, S. E. 1945 Mutations of bacterial viruses affecting their host range. Genetics 30:84–99. 17247148 10.1093/genetics/30.1.84 PMC1209277

[mgg3189-bib-0012] Mannucci, P. M. , and E. G. Tuddenham . 2001 The hemophilias–from royal genes to gene therapy. N. Engl. J. Med. 344:1773–1779. 11396445 10.1056/NEJM200106073442307

[mgg3189-bib-0013] McKusick, V. A. 1998 Mendelian inheritance in man. A catalog of human genes and genetic disorders, 12th ed Johns Hopkins Univ. Press, Baltimore, MD.

[mgg3189-bib-0014] Miller, I. , and D. D. Djkes . 1988 A simple salting out procedure for extracting DNA from human nucleated cells. Nucleic Acid Res. 16:1215–1220. 3344216 10.1093/nar/16.3.1215 PMC334765

[mgg3189-bib-0015] Morton, N. E. 1955 Sequential tests for the detection of linkage. Am. J. Hum. Genet. 7:277–318. 13258560 PMC1716611

[mgg3189-bib-0016] Reeve, J. P. , and B. Rannala . 2002 DMLE+: Bayesian linkage disequilibrium gene mapping. Bioinformatics 18:894–895. 12075030 10.1093/bioinformatics/18.6.894

[mgg3189-bib-0017] Sanchez‐Garcia, J. F. , D. Gallardo , L. Ramirez , and F. Vidal . 2005 Multiplex fluorescent analysis of four short tandem repeats for rapid haemophilia A molecular diagnosis. Thromb. Haemost. 94:1099–1103. 16363255 10.1160/TH05-05-0360

[mgg3189-bib-0018] Tantravahi, U. , V. V. Murty , S. C. Jhanwar , J. J. Toole , J. M. Woozney , R. S. Chaganti , et al. 1986 Physical mapping of the factor VIII gene proximal to two polymorphic DNA probes in human chromosome band Xq28: implications for factor VIII gene segregation analysis. Cytogenet. Cell Genet. 42:75–79. 3013509 10.1159/000132255

[mgg3189-bib-0019] Vehar, G. A. , B. Keyt , D. Eaton , H. Rodriguez , D. P. O'Brien , F. Rotblat , et al. 1984 Structure of human factor VIII. Nature 312:337–342. 6438527 10.1038/312337a0

[mgg3189-bib-0020] Winter, P. C. , H. Egan , O. McNulty , F. G. Jones , J. O'Donnell , and P. V. Jenkins . 2008 A recurrent F8 mutation in Irish haemophilia A patients: evidence for a founder effect. Haemophilia 14:394–395. 18179574 10.1111/j.1365-2516.2007.01639.x

